# Cutaneous leishmaniasis in a newly established treatment centre in the Lay Gayint district, Northwest Ethiopia

**DOI:** 10.1002/ski2.229

**Published:** 2023-03-17

**Authors:** Endalew Yizengaw, Endalkachew Nibret, Gizachew Yismaw, Bizuayehu Gashaw, Dessalegn Tamiru, Abaineh Munshea, Yegnasew Takele, Ingrid Müller, Lloyd Chapman, Richard Weller, James A. Cotton, Pascale Kropf

**Affiliations:** ^1^ Department of Medical Laboratory Science College of Medicine and Health Science Bahir Dar University Bahir Dar Ethiopia; ^2^ Institute of Biotechnology Bahir Dar University Bahir Dar Ethiopia; ^3^ Amhara Public Health Institute Bahir Dar Ethiopia; ^4^ Department of Biology College of Science Bahir Dar University Bahir Dar Ethiopia; ^5^ Nefas Mewcha Hospital Lay Gayint Ethiopia; ^6^ Department of Infectious Disease Imperial College London London UK; ^7^ Department of Dermatology University of Edinburgh Edinburgh UK; ^8^ Department of Infectious Disease and Epidemiology London School of Hygiene and Tropical Medicine London UK; ^9^ School of Biodiversity One Health and Veterinary Medicine Wellcome Centre for Integrative Parasitology College of Medical Veterinary and Life Sciences University of Glasgow Glasgow UK

## Abstract

**Background:**

Cutaneous leishmaniasis (CL) is a neglected tropical disease that primarily affects the most vulnerable populations. In Ethiopia, where this study took place, CL is an important health problem, however, the incidence of CL is poorly monitored.

**Objectives:**

This study took place in a recently established CL treatment centre, at Nefas Mewcha Hospital, Lay Gayint. This area was considered to be endemic for CL, however, no cases of CL from Lay Gayint had previously been officially reported to the Amhara Regional Health Bureau.

**Methods:**

Following a CL awareness campaign, a retrospective data review was performed of patients presenting to this centre between July 2019 and March 2021. Basic demographic and clinical data were collected by a nurse and recorded in the logbook of the CL treatment centre.

**Results:**

Two hundred and one patients presented for diagnosis and treatment. The age of the patients ranged from 2 to 75 years and 63.2% were males. Most patients were between 10‐ and 19‐years‐old. The majority (79.1%) of the patients presented with localised cutaneous leishmaniasis and 20.9% with mucocutaneous leishmaniasis. 98% of the patients tested positive for *Leishmania* parasites by microscopy.

**Conclusions:**

This work underpinned how CL is a major public health problem in the Lay Gayint district. It also shows that raising awareness about CL in the community and providing diagnosis and treatment encouraged patients to travel to seek diagnosis and treatment.

## Graphical abstract

Our work underpinned how cutaneous leishmaniasis (CL) is a major public health problem in the Lay Gayint district. It also shows that raising awareness about CL in the community and providing diagnosis and treatment encouraged patients to travel to seek diagnosis and treatment.

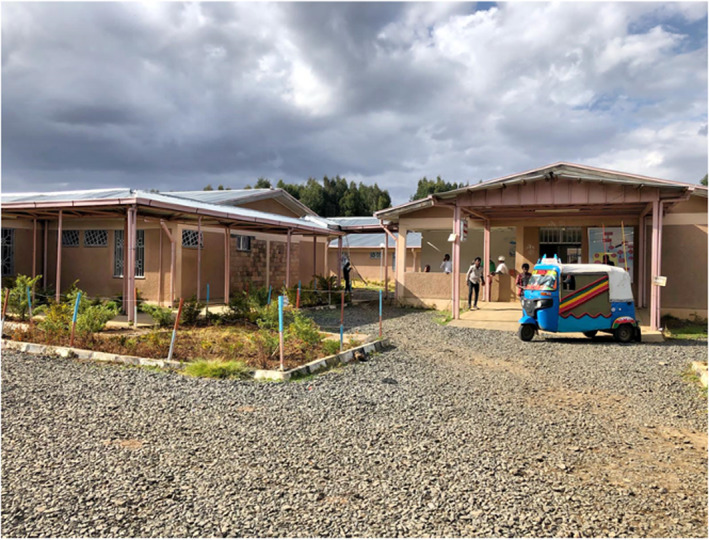

1

**What's already known about this topic?**

- Cutaneous leishmaniasis (CL) is one of the most overlooked of the neglected tropical diseases.
- In Ethiopia, 30 million people are living in areas endemic for CL but the prevalence of CL is still unknown.
- In Lay Gayint, where this study took place, no CL case were officially reported and there is no epidemiological information about the endemicity of CL in this district.

**What does this study add?**

- Our study is the first study describing the clinical prevalence and pattern of CL distribution in this remote and underserved area of Northwest Ethiopia.
- This work underpinned how CL is a major public health problem in Lay Gayint. It also shows that raising awareness about CL in the community and providing diagnosis and treatment encouraged patients to travel to seek diagnosis and treatment.

## 2 INTRODUCTION

Cutaneous leishmaniasis (CL) is a vector born protozoan disease endemic in 200 countries, with 253 435 new cases reported in 2018.^1^ There are three main clinical forms of CL in Ethiopia: localised CL (LCL), a disease characterised by single or multiple localised lesions on exposed areas of skin; mucocutaneous CL (MCL), affecting the mucosa of the nose and mouth; and diffuse CL (DCL), characterised by numerous non‐ulcerating nodules. LCL is the most frequent manifestation of CL. LCL usually heals spontaneously within 1 year but leaves unsightly scars. Persistent LCL, MCL and DCL require treatment and patients relapse frequently.^2^

Globally, there are more than 20 *Leishmania* species known to cause CL. While in the Old World, in the eastern hemisphere (Europe, Asia, and Africa) CL is predominantly caused by *Leishmania (L*.*) tropica*, *L*. *major*, and *L*. *aethiopica*; in the New World, in the western hemisphere (America) CL is mainly caused by *L*. *braziliensis*, *L*. *mexicana and L*. *amazonensis*.^3^

In Ethiopia, the vast majority (99.9%) of CL cases are caused by *L*. *aethiopica*, the remaining CL cases being caused by *L*. *tropica* and *L*. *major*.^4^, ^5^ Importantly, and in contrast to other endemic countries, MCL and DCL are relatively common in Ethiopia.^6^ CL in Ethiopia is more common in children, with the highest prevalence occurring between 10 and 15 years of age.^6^, ^7^, ^8^, ^9^

CL was first described in Ethiopia in 1913 and is present in most regions of Ethiopia.^10^ However, despite this wide distribution, CL is under‐reported and remains one of the most overlooked of the neglected tropical diseases in the country, predominantly affecting the most vulnerable populations.^11^ The exact prevalence is still unknown because of the lack of organised surveillance. This lack of knowledge of the extent of CL precludes effective control or prevention strategies.

CL is thought to be mainly transmitted zoonotically and rock hyraxes are thought to be the main reservoir hosts.^2^, ^6^ According to the Amhara Regional Health Bureau, about 80 areas are confirmed to be endemic for the disease. There are annually 20 000–30 000 reported CL cases in Ethiopia, with nearly 30 million people being at high risk.^12^

The Amhara National Regional State is one of the Ethiopian regions with a high burden of CL.^12^ According to the Amhara Regional Health Bureau, there are multiple CL endemic sites in Amhara Region such as Lay Gayint, Addis Zemen, Dega Damot, Finote Selam, Bibugn, Ankesha, Boru Meda, Dehana, and Kutaber. The Amhara Regional Health Bureau has estimated that in 2018, nearly 10 million people are at risk. However, due to the lack of surveillance and the remoteness of the areas where CL is endemic, only 4000–5000 cases are reported annually, which is likely to be a vast underestimation of the real burden of CL. And indeed, up to July 2019, no CL cases had been officially reported to the Amhara Regional Health Bureau by the health facilities of Lay Gayint. There is therefore no epidemiological information about the endemicity of CL in this district.

Following an awareness and training campaign among health extension workers (HEW) and health professionals in 2019, we set up a treatment centre at Nefas Mewcha in Lay Gayint, in collaboration with the Amhara Regional Health Bureau and the Ministry of Health. Before, the closest treatment centre was in Boru Meda, 231 km away from Nefas Mewcha.

The aim of this study was to make a preliminary assessment of the burden of disease in this remote and underserved region and analyse the pattern of CL disease in patients presenting to the treatment centre. This observational study will help to identify the burden of CL in the area and will be used as a baseline to conduct further molecular and epidemiological studies to understand the prevalence, spatial and temporal distribution of the disease, risk factors as well as the transmission dynamics.

## 3 MATERIALS AND METHODS

### 3.1 Study design

**FIGURE 1:**
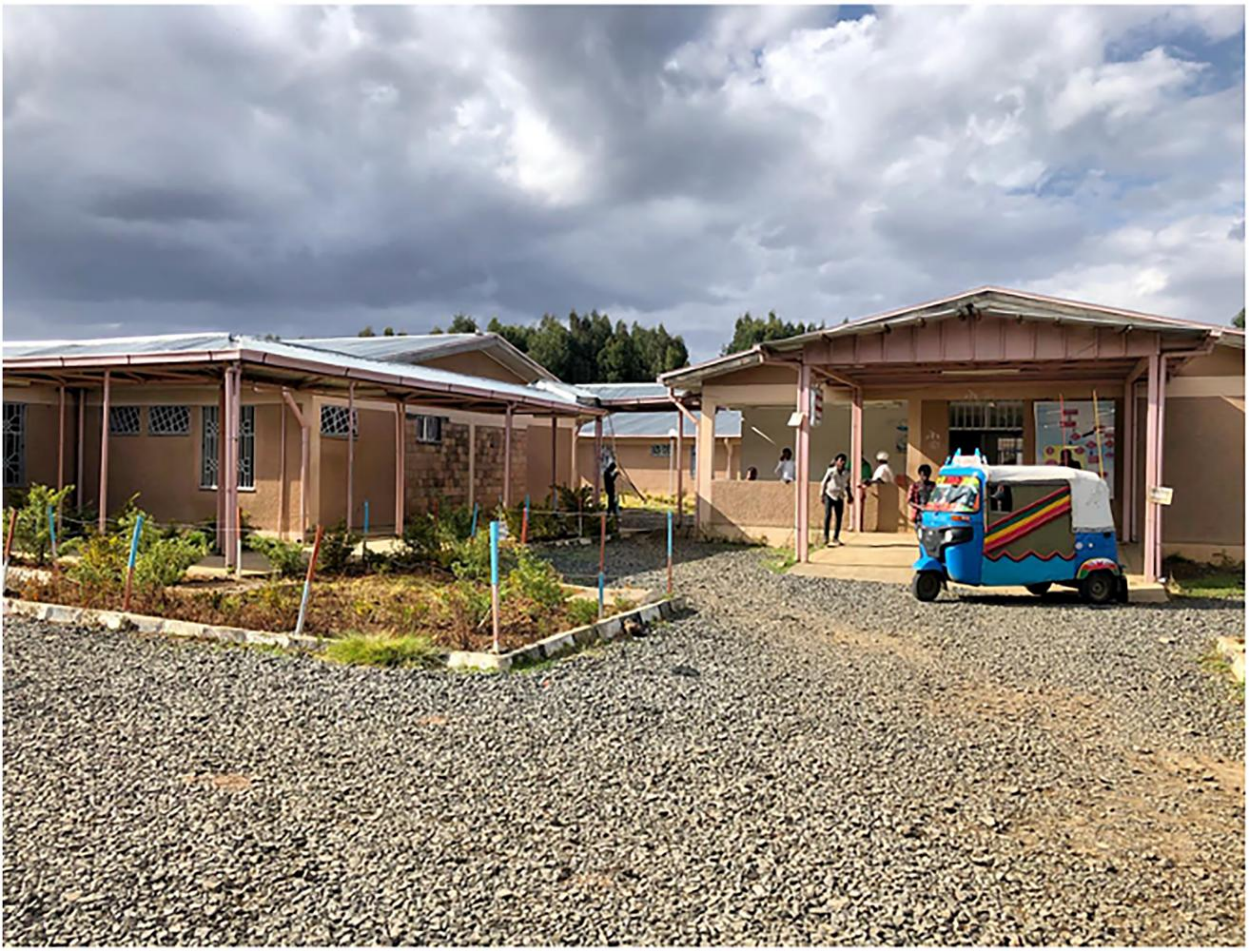
Nefas Mewcha Hospital.

**FIGURE 2:**
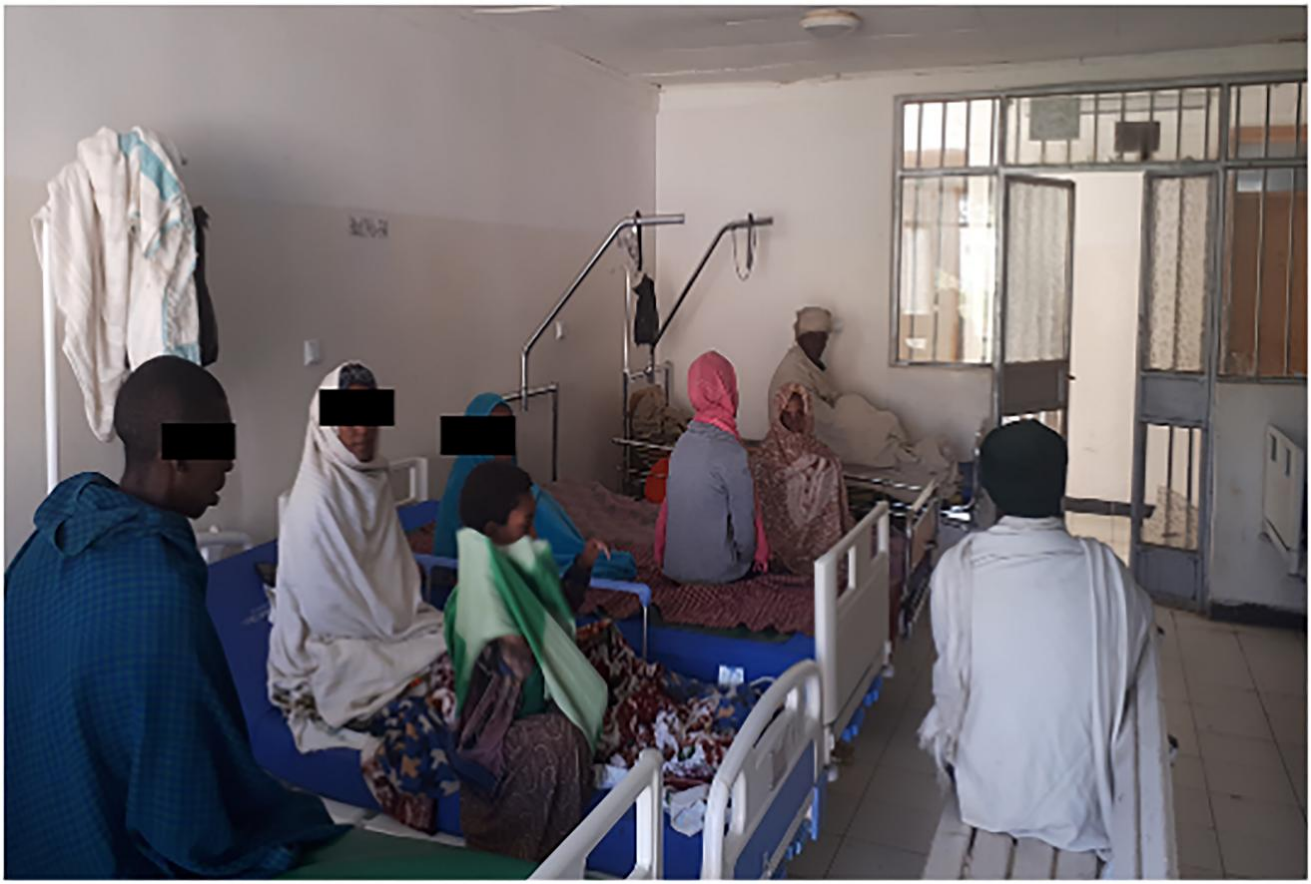
Cutaneous leishmaniasis patients in the Leishmaniasis Treatment Centre, Nefas Mewcha Hospital.

A retrospective review was done with CL patients who presented from July 2019 to March 2021 at Nefas Mewcha Hospital, a primary hospital in Lay Gayint, Northwest Ethiopia. Based on anecdotal evidence, an outbreak of CL was reported in Lay Gayint by the District Health Office in 2009. The diagnosis of CL was mainly based on the appearance of the lesions. However, no CL cases were officially reported to the Amhara Regional Health Bureau. Therefore, we first created awareness of the disease and its treatment to allow for identification of cases. Most preventive health care in Ethiopia is delivered by HEW. HEW are health professionals, two of whom are assigned to each Kebele (an administrative unit of around 1000 households and 5000 individuals) and visit every residence in their catchment area, providing health education and basic health services, while remaining embedded in the community. We mobilised over 100 HEWs and provided them with an extensive training programme on recognition and diagnosis of CL. Community representatives and hospital staff were also included in the training programme. In the week following this training, 321 potential CL patients were identified by HEW, based on the appearance of their skin lesions. Since the closest treatment centre was 5 h away by public transport, this high number of reported CL cases required the establishment of a local treatment centre in Nefas Mewcha hospital for all Kebele of Lay Gayint and surrounding districts. The Leishmaniasis Treatment Centre (LTC) at Nefas Mewcha hospital was thus established in 2019 in collaboration with the Ministry of Health, the Amhara Regional Health Bureau and the Amhara Public Health Institute (Figures 1 and 2). Health professionals were trained to diagnose CL and treat CL patients. Since then, the LTC has been playing a key role in the awareness of CL among health professionals and the local community.

### 3.2 Ethical approval and consent to participant

Ethical approval was secured from the Research and Ethical Review Committee of the College of Science (RECCS), Bahir Dar University (Ref RCSVD 002). Participant consent was waived by RECCS and the study participants were kept anonymous to maintain their medical confidentiality rights: Personal identifier variables like names were not included in the data collection checklist. The results of the study were reported to the host institution and Amhara Regional Health Bureau.

### 3.3 Study population and data collection

Individuals with potential CL lesions self‐presented to the LTC either on the recommendation of their HEW who had identified a lesion as potentially being CL; or on the patient having heard of the LTC by word of mouth. The Guidelines for diagnosis, Treatment and Prevention of Leishmaniasis in Ethiopia^12^ state that a ‘clinically suspicious lesion is defined as a skin nodule or ulcer with a raised edge appearing on someone who lives in an area known to be endemic for CL or visited such an area in the last 2 years’. To diagnose these individuals, a slit skin smear was collected from the edge of the lesion and stained with Giemsa.^12^ The diagnosis was confirmed by demonstration of amastigotes in the skin smear.

All confirmed CL cases were treated with sodium stibogluconate i.m. (20 mg/kg/day) for 28 days, as described in the Guidelines for diagnosis, treatment and prevention of leishmaniasis in Ethiopia.^12^

The data presented in this study represent all the information that are available from the nationally approved registration logbook of the LTC.

We collated the records of patients with CL who presented to the LTC. Data from the registration logbook were reviewed for basic demographic data, clinical forms of CL, skin smear microscopy result. This information was collected using a structured data collection questionnaire developed for this purpose.

### 3.4 Statistical analysis

The data was entered and analysed using Statistical Package for Social Science 23 (SPSS‐23), except the Fisher's exact test for contingency tables larger than 2 × 2, which was performed in R v4.0.2, with *p*‐values estimated using 100 000 Monte Carlo simulations. A Mann‐Whitney test (Prism 9) was used to assess statistical difference between the age of females and males. Differences were considered statistically significant at *p* < 0.05. *p < 0.05, **p < 0.01, ***p < 0.001 and ****p < 0.0001. Unless otherwise specified, results are expressed as median ± SEM.

## 4 RESULTS

### 4.1 Socio‐demographic characteristics

A total of 201 CL patients presented at the LTC in Nefas Mewcha Hospital for diagnosis and treatment from January 2019 to August 2021. As shown in Table 1, 71 individuals were diagnosed for CL in 2019, 95 in 2020 and 34 in 2021, the date was not recorded for one individual. The sex distribution was 63.2% male and 36.8% female. The age of the CL patients ranged from 2 to 75 years with a median age of 18 ± 1 year. There was no significant difference in age between female and male patients (15.0 ± 1.8 and 20.0 ± 1.1, *p* = 0.0550, data not shown).

**TABLE 1:** Number of patients diagnosed per month.

	Jan	Feb	Mar	Apr	May	Jun	Jul	Aug	Sept	Oct	Nov	Dec	Total
2019	1			1		6	3	1	8	21	6	24	71
2020	6	7	12	10	14	3	7	7	3	8	3	15	95
2021	11	7	5	1	7			3					34

*Note*: The number of patients diagnosed for CL at the LTC in Nefas Mewcha Hospital for diagnosis and treatment from January 2019 to August 2021 were recorded per month.

Abbreviations: CL, cutaneous leishmaniasis; LTC, Leishmaniasis Treatment Centre.

The highest numbers of CL cases (female: *n* = 32 and male: *n* = 44) presenting at the LTC during the study period belong to the 10–19 years age group (Table 2).

**TABLE 2:** CL cases amongst the Lay Gayint population.

Age groups (years)	Lay Gayint population		CL case	
	Female	Male	Female (%)	Male (%)
0–9	28 701	29 681	14 (0.05)	18 (0.06)
10–19	26 606	28 525	32 (0.12)	44 (0.15)
20–29	16 328	15 156	13 (0.08)	34 (0.22)
30–39	10 980	11 353	4 (0.04)	8 (0.07)
40–49	8711	7134	6 (0.07)	10 (0.14)
50–59	6126	6803	1 (0.02)	6 (0.09)
60–69	4811	4157	3 (0.06)	4 (0.10)
70–79	1507	2804	1 (0.07)	3 (0.11)
≤80	816	1276	0	0
Total	104 586	106 889	74 (0.07)	127 (0.12)

*Note*: The number of males and females per age group in Lay Gayint in 2021 was obtained from the Amhara National Regional State Bureau of Finance.

Abbreviation: CL, cutaneous leishmaniasis.

The number of females and males per age group in Lay Gayint was obtained from the Amhara National Regional State Bureau of Finance; to assess the percentages of females and males who presented to the LTC with CL. The number and percentage of CL cases per age group is shown in Table 2. The highest percentage of CL cases amongst female patients was in the 10–19 age group (0.12%), and for male patients it was in the 20–29 age group (0.22%). Age group was significantly associated with the rate of CL presentation in males (Fisher's exact test *p* = 0.0002) and in the total population (Fisher's exact test *p*∼1 × 10^−5^) but was not significant in females (Fisher's exact test *p* = 0.055). The number of CL cases in females was lower than those in males in all age groups (Table 2) and the proportion of the female population reporting to the clinic with CL was significantly lower than in the male population (odds ratio 0.59; 95% CI for odds ratio 0.44–0.79; Fisher's exact test *p* = 0.0004).

### 4.2 CL lesion types

Most patients (79.1%) had LCL lesions while 20.9% had MCL lesions. One hundred and ninety‐seven patients (98%) tested positive for CL on microscopy and clinical signs. Four further patients were considered to have a positive diagnosis despite being negative on microscopy based on the appearance of their lesions (2 MCL and 2 LCL, data not shown). All 201 patients were treated with sodium stibogluconate i.m. (20 mg/kg/day) for 28 days.^12^ Of these 201 patients, 5 (2.5%) had already received an anti‐leishmanial treatment in another treatment centre.

## 5 DISCUSSION

This is the first study describing the clinical prevalence and pattern of CL distribution in this remote and underserved area of Northwest Ethiopia. Over a 20‐month period, 201 incident cases of CL were positively identified based on clinical features and parasitological confirmation. This would suggest a crude annual incidence rate of parasitologically proven active CL in Lay Gayint of around 6 in 10 000 but we believe this is an under‐reporting of the true incidence. Patients who were positively diagnosed had to travel long distances and then commit to at least a 1‐month period of treatment after diagnosis.^12^ Previous studies of active CL prevalence in Ethiopia have produced figures ranging from 0.01% to 10.8%^6^ but these have not been random sampling and were biased by the selection of communities where outbreaks had been reported.^6^ We believe that our data are more likely to represent the endemic background disease prevalence in highland Ethiopia, as our treatment centre served a large, stable and hitherto unserved region. Prior to the establishment of the LTC no CL cases were officially reported by the health facilities in Lay Gayint and therefore CL was not considered a major public health problem. In rural Ethiopia patients have either assume no treatments were available, and left CL lesions untreated, used traditional local treatments, or took advice from religious leaders on treatment. Our experience underlines a significant unmet clinical need but also the encouraging observation that patients will seek treatment when they find out that a treatment centre exists. This clearly illustrates that CL is neglected and under‐reported in this area. These real‐world data on prevalence and the information this gives on social and economic consequences is important for the planning and establishment of appropriate control, treatment, and prevention strategies. It is also important to note that the evidence‐base for treatment of CL caused by *L*. *aethiopica* is particularly limited, and that more studies are needed to establish what treatments are best and most importantly explore alternative lines of treatments.^13^

In agreement with other studies both in Ethiopia^9^, ^14^, ^15^ and more widely in other countries such as Iran^16^, ^17^ and Yemen,^18^ more males were diagnosed (63.2%) with CL.^3^ This may represent different work patterns, with men more likely to be outside and thus exposed to sand fly vectors. Males are usually responsible for agricultural activities, sleeping in temporary shelters in crop areas and looking after animals in the field. Behavioural factors may also play a part. In Bolivia it has been shown that males who spend more time outdoor in late evenings have an increased risk to be bitten by sand flies.^19^ Selection bias cannot be discounted; the higher number of male patients presenting at the LTC might also indicate that males are more likely to travel to distant hospitals for diagnosis and treatment than women.

The majority of CL patients were in the 10–29 year‐old age band, but with females having a peak incidence in the 10–19 age band and males in the 20–29 band. This may represent selection bias with people in this age group more likely to seek treatment, although we consider this unlikely. These are peak educational and working years, with the highest opportunity cost for visiting hospital, getting treatment, and being away from productive activity. We suspect that behavioural or immunological factors account for this age distribution. Women in the 20–29 age group stay more indoors as compared to men in the same age group, as men are mostly engaged in agricultural activities and sleep outdoors in temporary shelters on the farms. Social, religious and cultural activities also occur predominantly outside. This predominantly outdoor living brings CL patients, rock hyrax colonies and sand fly vectors into closer proximity and increases the chance of the transmission of the parasite. An alternative explanation may be the development of immunity to CL with previous exposure. Prospective data show this to occur with *L*. *infantum* in Iran^20^ and suggest that protective anti‐leishmania vaccines are achievable.^21^

CL patients can present with different clinical forms of the disease. LCL is characterised by a single or multiple lesions on the exposed parts of the body. MCL affects the mucosa of the nose and mouth, whereas DCL is manifested in different parts of the body with non‐ulcerative nodular lesions. In this study, most of CL patients presented with LCL. LCL is the most common form of CL worldwide and it is also the case in Ethiopia.^6^ Unlike any other CL endemic areas of the Old World, MCL manifestation is unusually high (20.9%) in this study.^22^ Similar results (19.2%) have been reported from the southern part of Ethiopia.^23^ And a retrospective study conducted in Gondar between 2014 and 2015 also showed that 67 of 178 CL cases were MCL cases.^6^, ^8^ We cannot exclude that this high number of MCL cases is not representative of the frequency of MCL in this area; indeed, it is possible that due to the nature of the lesion, MCL patients tend to present more readily to the treatment centres. Further studies are needed to unravel the causes for the increased prevalence of CL patients presenting with MCL. No DCL patients presented to the centre during the time of this study; this might be explained by the fact that DCL is rare in Ethiopia^6^ or that because DCL is particularly disfiguring patients felt too stigmatised to seek help.

This work underpinned how CL is a major public health problem in the Lay Gayint district. It also shows that raising awareness about CL in the community and providing diagnosis and treatment encouraged patients to travel to seek diagnosis and treatment in the new LTC. Further studies for effective prevention and treatment are needed as CL can cause permanent scarring and disfigurement, that result in stigmatisation of the affected persons in the community. In addition to the stigma, the significant financial burden associated to the reduced ability of CL patients to work and support their family should also be considered.

## CONFLICT OF INTEREST STATEMENT

The authors have declared that no conflict of interest exists.

## AUTHOR CONTRIBUTIONS

**Endalew Yizengaw**: Conceptualization; Data curation; Formal analysis; Investigation; Project administration; Writing – original draft; Writing – review & editing. **Endalkachew Nibret**: Formal analysis; Writing – review & editing. **Gizachew Yismaw**: Formal analysis; Investigation. **Bizuayehu Gashaw**: Conceptualization; Data curation; Formal analysis; Investigation. **Dessalegn Tamiru**: Formal analysis. **Abaineh Munshea**: Formal analysis. **Yegnasew Takele**: Formal analysis; Investigation; Writing – review & editing. **Ingrid Müller**: Formal analysis; Funding acquisition; Investigation; Writing – original draft; Writing – review & editing. **Lloyd Chapman**: Formal analysis; Funding acquisition; Investigation; Methodology; Writing – original draft; Writing – review & editing. **Richard Weller**: Formal analysis; Investigation; Writing – original draft; Writing – review & editing. **James A. Cotton**: Formal analysis; Funding acquisition; Investigation; Methodology; Writing – original draft; Writing – review & editing. **Pascale Kropf**: Data curation; Formal analysis; Funding acquisition; Investigation; Supervision; Writing – original draft; Writing – review & editing.

## ETHICS STATEMENT

Ethical approval was secured from the Research and Ethical Review Committee of the College of Science (RECCS), Bahir Dar University (Ref RCSVD 002). Participant consent was waived by RECCS and the study participants were kept anonymous to maintain their medical confidentiality rights: Personal identifier variables like names were not included in the data collection checklist. The results of the study were reported to the host institution and Amhara Regional Health Bureau.

## Data Availability

The data that support the findings of this study are available on request from the corresponding author. The data are not publicly available due to privacy or ethical restrictions.
